# The Schnitzler syndrome

**DOI:** 10.1186/1750-1172-5-38

**Published:** 2010-12-08

**Authors:** Dan Lipsker

**Affiliations:** 1 Clinique Dermatologique, Faculté de Médecine, Université de Strasbourg and Hôpitaux Universitaires, 1, place de l'hôpital, F-67091 Strasbourg cedex, France and INSERM U725 Biology of Human Dendritic cells

## Abstract

The Schnitzler syndrome is a rare and underdiagnosed entity which is considered today as being a paradigm of an acquired/late onset auto-inflammatory disease. It associates a chronic urticarial skin rash, corresponding from the clinico-pathological viewpoint to a neutrophilic urticarial dermatosis, a monoclonal IgM component and at least 2 of the following signs: fever, joint and/or bone pain, enlarged lymph nodes, spleen and/or liver, increased ESR, increased neutrophil count, abnormal bone imaging findings. It is a chronic disease with only one known case of spontaneous remission. Except of the severe alteration of quality of life related mainly to the rash, fever and pain, complications include severe inflammatory anemia and AA amyloidosis. About 20% of patients will develop a lymphoproliferative disorder, mainly Waldenström disease and lymphoma, a percentage close to other patients with IgM MGUS. It was exceedingly difficult to treat patients with this syndrome until the IL-1 receptor antagonist anakinra became available. Anakinra allows a complete control of all signs within hours after the first injection, but patients need continuous treatment with daily injections.

In many aspects, the Schnitzler syndrome resembles the genetically determined auto-inflammatory syndromes involving activating mutations of the NLRP3 inflammasome. This latter point and its consequences will be addressed.

## Background

The Schnitzler syndrome is a rare and acquired systemic disease which bears in common many features with a group of inherited diseases referred to as auto-inflammatory syndromes. Its main clinical features include fever, an urticarial rash, muscle, bone and/or joint pain and enlarged lymph nodes. A monoclonal IgM component is the biological hallmark of the disease. Conventional therapies including anti-histamines for the skin rash, as well as anti-inflammatory drugs, steroids and immunosuppressive drugs for the systemic signs, are usually ineffective. However, the IL-1 receptor antagonist anakinra was found to rapidly control all the symptoms of this syndrome. However, signs recur as soon as the treatment is stopped. About 15% to 20% of patients with a Schnitzler's will develop a lymphoproliferative disorder, a prevalence shared with other patients with monoclonal IgM gammopathies of undetermined significance (MGUS) [1]. AA-amyloidosis is a concern in untreated patients [2,3].

This review will provide a comprehensive overview of the clinical and biological features of this syndrome, emphasizing its particular rash, and summarize our current comprehension of its pathophysiology, its complications and its treatment.

### History

The different signs of this syndrome were first reported in 1972 and then published in 1974 as an autonomous entity by Liliane Schnitzler, a French dermatologist [4,5]. In the following years, cases were reported from all over the world including North America and Japan, but mostly from Europe. The European preeminence is probably related to a better knowledge of this entity in the old World. In 1999, Lipsker et al reported 4 cases and performed an extensive literature review which allowed them to establish diagnostic criteria [6], which are currently accepted [3]. In their paper, they included the CINCA (Chronic infantile Neurological Cutaneous and Articular syndrome)/NOMID (Neonatal Onset Multi-Inflammatory Disease) and the Muckle-Wells syndrome in the differential diagnosis and thus pointed for the first time to similarities between the Schnitzler syndrome and the auto-inflammatory syndromes, of which the latter are a paradigm. Indeed, the CINCA syndrome, the Muckle-Wells syndrome and familial cold auto-inflammatory syndrome are different phenotypes of the cryopyrinopathies, monogenic diseases involving the innate immune system. Their pathophysiology implies exaggerate activation of the inflammasome, an IL-1 synthesizing cellular machinery [7]. And indeed, IL-1 inhibition is a very effective treatment modality in patients with CINCA. Since the Schnitzler syndrome shares many features with the CINCA syndrome, anakinra, an IL-1 inhibitor was also tried in the former syndrome. It proved to be the first really efficient treatment of the Schnitzler syndrome.

### Clinical Findings

The Schnitzler syndrome is characterized by a recurrent febrile rash, joint and/or bone pain, enlarged lymph nodes, fatigue, a monoclonal IgM component, leucocytosis and systemic inflammatory response. The reviews performed by Lipsker et al in 1999 and de Koning et al in 2007 summarize most published cases [3,6]. They form the basis of this review, to which the author's own experience with more than 10 patients, as well as recent publications, is added. Diagnosis can be established when the criteria summarized in Table 1 are met.

**Table 1:** Diagnostic criteria of the Schnitzler syndrome

Urticarial skin rash^1,2 ^and monoclonal IgM component^3 ^and at least 2 of the following criteria^4^:
Fever

Arthralgia or arthritis

Bone pain

Palpable lymph nodes

Liver or spleen enlargement

Elevated ESR

Leukocytosis

Abnormal findings on bone morphologic investigations

Another cause must be eliminated in all cases (see differential diagnosis), most notably hyper IgD syndrome, adult onset Still disease, urticarial hypocomplementemic vasculitis, acquired C1 inhibitor deficiency and cryoglobulinemia.

^1^: the urticarial skin rash has now been extensively described and corresponds nosologically to a neutrophilic urticarial dermatosis [9]

^2^: some authors have reported patients with all signs of the Schnitzler but without the typical skin rash [8]; though those patients have probably a very similar disease, until further characterization, the peculiar skin rash should remain an obligate diagnostic criteria.

^3^: Some patients have a monoclonal IgG component instead of a monoclonal IgM [reviewed in [3]]

^4^: In patients treated with IL-1 inhibitors, a rapid and immediate response is supportive of the diagnosis. In case of unresponsiveness to anakinra, the diagnosis should be reconsidered.

#### Epidemiology

There is a slight male predominance and mean age of disease onset is 51 years. The youngest patient reported started urticaria at age of 13, but the Schnitzler syndrome is basically a disease of the adult, since only four patients started disease before age of 35 [3]. The delay to diagnosis exceeds 5 years in many cases.

#### The skin rash

As the skin rash - with the monoclonal component - is a defining criteria of the syndrome, it is present *by definition *in all patients. Patients with all signs of the Schnitzler syndrome except the skin rash should be referred to as Schnitzler-like syndrome [8].

**Figure 1:**
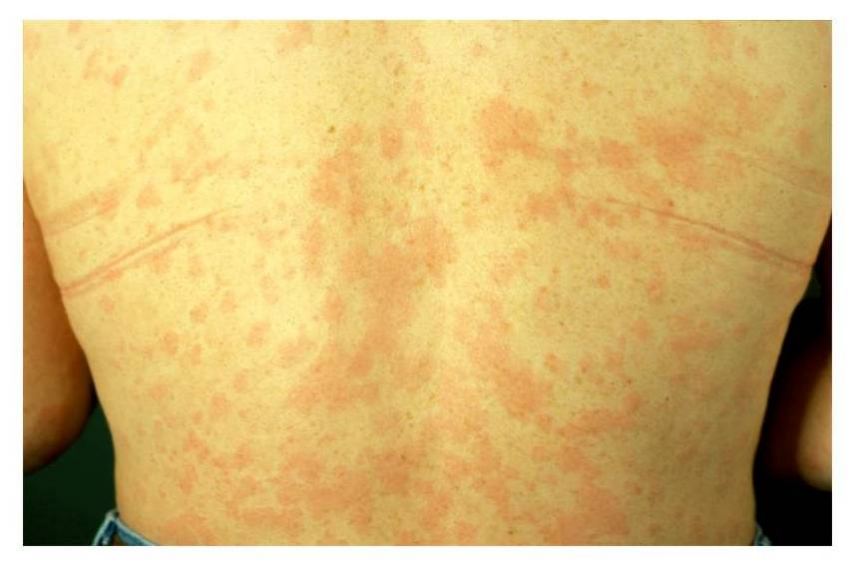
**the typical rash of the Schnitzler syndrome, which corresponds to a neutrophilic urticarial dermatosis: rose to red macules and/or slightly raised plaques**. The lesions are usually not itchy and vanish within hours without sequel.

The skin rash is usually the first clinical sign and most patients started their disease with the eruption. The skin rash was classically referred to as "urticaria". However, recently this peculiar rash was described in detail and nosologically delineated from common urticaria [9]. Patients with the Schnitzler syndrome have a rose pale or red eruption consisting of macules (flat lesions) or slightly raised papules and plaques (Figure 1). They last less than 24 hours, and are usually not or only moderately itchy. Lesions can occur on every body part, though involvement of face and extremities is rare. Edematous swelling of the face (angioedema) is very rare [3,10,11] and significant mucosal swelling with dyspnea and/or dysphonia is exceptional. Many patients report exacerbating factors including heat exposure, cold-exposure, alcohol consumption, some aliments, physical work, stress,... There is no specific chronology of the rash, as would be typical for example in patients with adult onset Still disease (AOSD) who report flares in the late afternoon. Confluence of lesions is possible, as is dermographism, i.e. friction of skin induces slightly raised plaques, though they are usually not itchy as they would be in authentic dermographism. The frequency of flares is variable from patient to patient, and in the same patient from factors we yet ignore. Patients can have daily flares for months or years or remission for days to weeks. It is however exceedingly rare to be free of rash for a period longer than a month in untreated patients. Skin lesions resolve within hours without any sequel.

**Figure 2:**
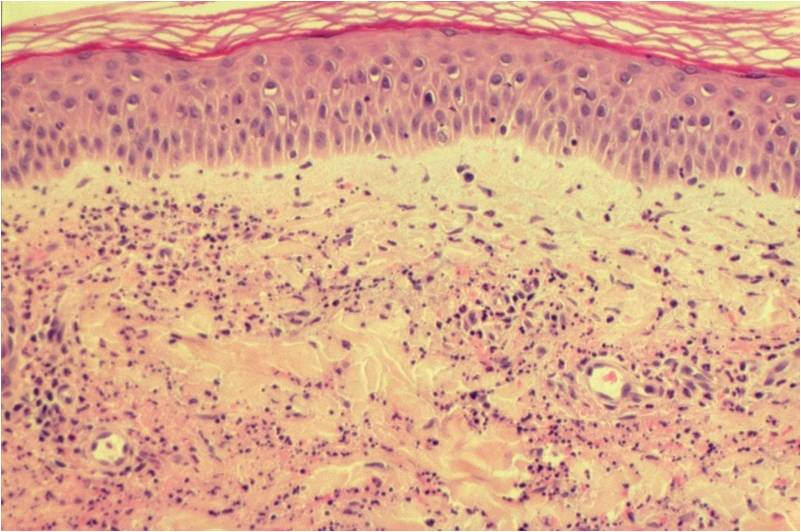
**(HE, × 200): a neutrophilic infiltrate of the dermis without vasculitis and without significant edema**.

**Figure 3:**
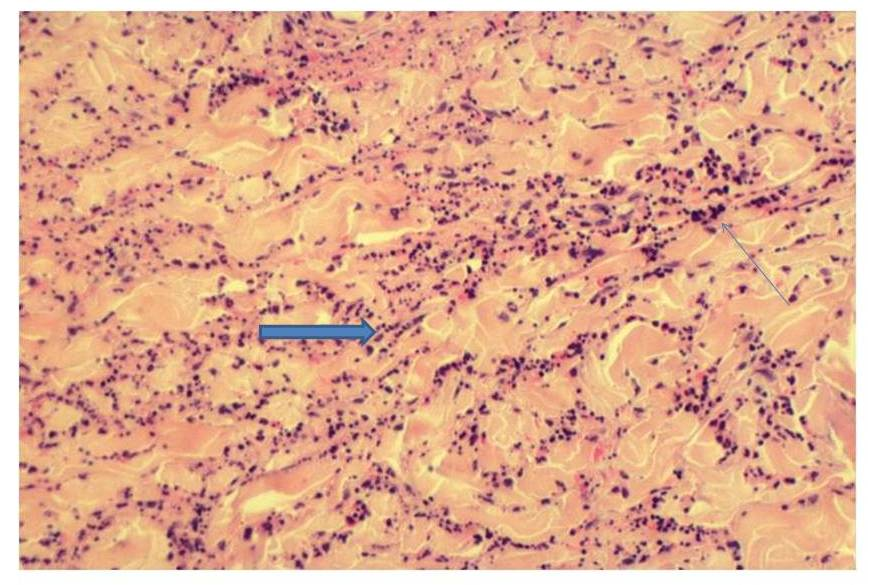
**(HE, × 200): significant neutrophilic infiltrate (arrow, thin) with interstitial dispersion and leukocytoclasia (arrow, thick)**.

The histopathological findings are noteworthy [9,12]. When a typical plaque is biopsied at a relatively early stage, a neutrophilic infiltrate of the dermis can be found (Figure 2). The density of the infiltrate is variable. Interstitial dispersion with neutrophils along the collagen bundles is typical, as is significant leukocytoclasia (Figure 3). There are no vasculitis and no significant dermal edema, which allows distinction from urticarial vasculitis and the Sweet syndrome respectively. The epidermis is usually normal; hypodermal involvement with neutrophils in the septa can be found, as can clustering of neutrophils around sweat ducts realizing a histopathologic aspect of eccrine hidradenitis as an epiphenomenon. Vasculitis has been reported in up to 20% of patients [3]. However, this author had the occasion to review 4 biopsies of patients with the Schnitzler syndrome previously interpreted as vasculitis, but in which clear-cut signs of vasculitis were lacking and which were re-interpreted as neutrophilic urticarial dermatosis. Furthermore, though published photographies of skin biopsies are rare, their careful examination rarely shows authentic vasculitis. For example, the picture published by Tanneberger et al and interpreted as vasculitis lack the typical features of the latter, as there is no fibrinoid necrosis of vessel wall [13].

When immunofluorescence studies were performed, deposition of immunoreactants, mainly IgM, could be found around the superficial dermal vessels in about 30% of the patients [6,14-18]. In a few interesting observations, IgM deposits were found along the dermal-epidermal junction [19-21]. It was demonstrated by means of immunoelectron microscopic studies and immunoblotting on epidermal and dermal skin extracts, that IgM-skin interactions occur [22]. Indeed, anti-skin IgM autoantibodies of the same isotype as their monoclonal gammopathies can be present in the serum of some patients with the Schnitzler syndrome. These IgM autoantibodies seem to deposit *in vivo *in the epidermis and at the dermal-epidermal junction, in the region of the anchoring fibrils. Their exact significance remains unclear, though it was postulated that they could trigger a local inflammatory response that could induce the skin lesions [22].

This rash, urticarial clinically, and a neutrophilic dermatosis histopathologically was recently delineated under the designation neutrophilic urticarial dermatosis (NUD) [9]. NUD can also occur in patients with AOSD, lupus erythematosus and cryopyrinopathies, which should be considered in the differential diagnosis of the Schnitzler syndrome [9].

#### The fever

Almost all patients will develop fever. The fever is intermittent. Body temperature can rise above 40°C. Most patients manage the fever and due its recurrent nature, get used to it. The fever is usually well tolerated and chills are rare. Fatigue is however frequent during the fever flares. In most patients, there is no relation between the fever and the skin rash. Fever responds in some patients to NSAI drugs and/or to steroids and is usually completely controlled with anakinra [6,23].

#### Musculoskelettal involvement

Musculoskelettal involvement is another cardinal feature of the disease affecting about 80% of patients. Bone pain is the most characteristic finding, but arthralgias and sometimes fully developed arthritis, can occur. Joint destruction and/or deformities have not been reported so far. Bone pain affects mostly the iliac bone and the tibia. Femur, spine, forearm and clavicle were less often involved [24]. Bone involvement is common, and 30 to 40% of the patients showed bone lesions on imaging studies [25]. Osteocondensation is the most frequent radiological finding and the most common radiological pattern is a sclerotic bone marrow involvement with cortical hyperostosis of distal femora and proximal tibiae, and a lack of suggestive signs of malignancy. Lytic lesions were reported [26,27] as well as periosteal apposition [24,28]. Radiological differential diagnosis is broad and includes mastocytosis, POEMS syndrome, Erdheim-Chester disease, Camurati-Englemann or Van Buchem disease, Buschke-Ollendorf syndrome, osteopetrosis, melorheostosis, ribbing disease and hypertrophic osteoarthropathy. Bone technetium scanning reveals hyperfixation in the areas of radiological involvement [24]. Magnetic resonance imaging confirms thickening of cortices and can show medullar bone involvement and marrow infiltration without space occupying features in the affected areas [24,28,29]. A solitary sclerotic lesion has been reported [30] and thus radiological differential diagnosis should be expanded to include entities as lymphoma, multiple myeloma, SAPHO syndrome, osteoblastic metastases, osteosarcoma, chronic osteomyelitis, osteoid osteoma, and healing stress fractures [31]. Bone biopsy of involved areas can be normal [32,33] or show an unspecific inflammation in 5 patients [34-38], sometimes associated with hyperactive osteoblasts. One patient had histological evidence of osteosclerosis [21]. Heterotopic ossification after total hip arthroplasty has been reported [39].

#### Organomegaly

*Palpable lymph nodes *are found in about 45% of the patients and *hepatic or splenic enlargement *occurs in about a third of the patients [3,6]. Palpable lymph nodes are found in the axilla and inguinal sites and sometimes in the cervical region. Those lymph nodes can be multiple, permanent and up to 2 or 3 cm large and therefore suggest the diagnosis of lymphoma, but biopsy shows non-specific inflammation.

### Biological Findings

#### Monoclonal component

The *monoclonal IgM component *is a defining feature of the syndrome and thus present in all patients [6]. Patients with all signs of the Schnitzler syndrome except the monoclonal component should be referred to as Schnitzler-like syndrome.

In more than 90% of patients, the monoclonal IgM gammopathy is associated to a kappa light chain. Usually, when diagnosis of the syndrome is made, IgM levels are low (< 10 g/L in 67% of the patients). IgM levels can remain stable or progressively increase at a rate of about 0.5 to 1 g/L/year. High IgM levels should raise suspicion of Waldenströms disease. When patients are seen at the very beginning of the disease, monoclonal IgM component can be present at very low (trace) level (Lipsker, personnal observation). There are some reports of Schnitzler syndromes (< 10% of reported cases) with an associated IgG monoclonal component [6,40] and even one report of a 58-year-old woman displaying many clinical features of the syndrome and a polyclonal increase in IgG and IgA [41]. Bence-Jones proteinuria was reported in about 30% of the patients. In about 25% of the patients, lowered levels of IgG or IgA can be found [6]. At time of diagnosis, examination of bone marrow is normal in 80% of the patients. In the remaining 20%, unspecific, polyclonal, lymphocytic or plasmocytic infiltrates were reported.

#### Other biological findings

During the course of the disease, *elevated ESR *is a constant feature. *Complement levels *are normal or increased in patients with this syndrome. When complement levels are lowered, another diagnosis must be considered, especially hypocomplementic urticarial vasculitis and cryoglobulinemia, and the possibility of a genetic deficiency of C4 should be addressed, since 2 patients with Schnitzler syndrome had a C4a deficiency [42]. *An inflammatory anemia*, and sometimes *thrombocytosis*, is present in up to 50% of the patients. Inflammatory anemia can be very severe and symptomatic [6,15]. Persistent *leukocytosis of neutrophils *(> 10 × 10^9^/L), in the absence of any treatment, occurs in more than 2/3 of the patients [3,6]. This is a relevant finding in this clinical context, as most auto-immune differential diagnoses (urticarial vasculitis, cryoglobulinemia) are not associated with increased number of neutrophils. In this regard, both, adult onset Still's disease and the Schnitzler syndrome can associate a skin rash, fever, palpable lymph nodes, spleen and liver enlargement, arthralgias and leukocytosis [43]. However, ferritine levels are usually more elevated in the former while a monoclonal IgM component is present in the latter.

### Associated Findings

Associated findings include pseudoxanthum elasticum in 2 patients [44,45], peripheral neuropathy with the presence of monoclonal IgM with anti-MAG (myelin-associated glycoprotein) in 1 patient [46], C4 deficiency in 2 patients [42] and nodular regenerative hyperplasia of the liver in 1 patient [47], impairment of renal function in 1 patient [48], thrombophilia with anti-phospholipid antibodies and hyperhomocysteinemia [49] and hearing loss [3]. This latter finding is especially interesting as it is also found in the genetic auto-inflammatory diseases Muckle-Wells and CINCA syndromes. The fact that hearing loss resolved with anakinra further supports strong similarities between the Schnitzler syndrome and the latter group of diseases. This author is aware of another patient with a monoclonal IgM component and late-onset symptoms of Schnitzler syndrome and hearing loss, with a rash triggered by cold-exposure (Prof. J.-H. Saurat, personal communication).

### Disease Course and Complications

The course of the disease is longstanding. Spontaneous or treatment-induced remissions have not been published. At least 3 patients developed inflammatory AA amyloidosis [2,3], a serious complication. However, though patients have a longstanding course with a monoclonal IgM component, no patient with systemic AL amyloidosis has yet been reported. Nevertheless, AL-amyloidosis should be considered as a possible complication of the syndrome.

The overall prognosis of the Schnitzler syndrome depends on the possible evolution into a lymphoproliferative disorder, either lymphoma, including lymphoplasmacytic lymphoma, lymphoma of the Richter type, marginal zone lymphoma, IgM myeloma or Waldenström's disease [42,50-56]. About nineteen percent of the reported patients with this syndrome developed lymphoproliferative disorders [3,6], a percentage close to the 18% prevalence at 10 years of lymphoproliferative disorders in patients with IgM MGUS in general [1]. However, the number of patients with the syndrome who will eventually develop lymphoproliferative disorder could be higher, since most patients were published shortly after diagnosis and therefore follow-up was too short to draw any conclusion about long-term outcome. Lymphoma or Waldenström's disease appears more than 10 to 20 years after the beginning of the first signs of the syndrome in most cases. Schnitzler's original patient died from diffuse lymphoplasmacytic infiltration of the liver and bone marrow 23 years after the beginning of the disease [50]. In rare cases, Waldenström's disease was revealed by a Schnitzler syndrome [17]. There is no specific predictive factor of the development of a lymphoproliferative disorder. Thus, initial work-up of a patient with this syndrome should include an examination of bone marrow, immunoelectrophoresis of seric and urinary proteins, and dosage of immunoglobulin subtypes. The two latter examinations can then be used to follow-up those patients on a biannual basis. Lymph nodes should be biopsied when they enlarge.

This author is aware of one patient with a typical Schnitzler syndrome which evolved for more than 8 years and then went into remission, without medical treatment at this time [patient 2 in reference [6]]. The remission now lasts for more than 5 years. The patient has only exceptional cold- or stress- triggered crisis once or twice a year. The monoclonal component is still present at more than 30 g/L. To the best of this author's knowledge, this is the only patient who went into spontaneous remission.

### Differential Diagnosis and Diagnostic Criteria

Diagnostic criteria are shown in Table 1. There is no specific biological marker for this disease. Thus, diagnosis relies on a combination of clinical, biological and radiological findings as well as on exclusion of another cause. Especially, the following diseases/entities need to be excluded: cryoglobulinemia, hypocomplementic urticarial vasculitis, acquired C1 inhibitor deficiency, hyper IgD syndrome and adult onset Still's disease. Furthermore, a spectacular and immediate response to anakinra is another finding that supports the diagnosis, as already suggested by Gilson et al. [57].

### Pathophysiology

The pathophysiology of the fever and of the syndrome in general remains unclear. Previous studies showed disturbed cytokine balance. The presence of anti-IL-1 antibodies was reported with increased frequency in this syndrome by Saurat et al [58], but this finding was subsequently not confirmed by other investigators [18,21,36,59]. We found elevated IL-6 and/or IL-2 receptor levels in some patients and normal TNFα and IL-8 levels (**6**). Although IL-6 is an essential plasma cell growth factor, it is also an acute phase reactant and its increase during a systemic illness is therefore not surprising. Previous studies suggested that IgM deposits in the skin seem to be involved in the pathophysiology of the rash [22].

The main unresolved question is whether the clonal IgM proliferation is primitive in nature or the result of a continuous antigenic stimulation. Thus, the question whether the Schnitzler syndrome is a smoldering lymphoproliferative disorder with systemic expression, comparable to the POEMS syndrome, or a systemic disorder with an accompanying mIgM remains open. In rare instances, treatment of the underlying lymphoproliferative disorder had a beneficial effect on the Schnitzler syndrome, supporting the former point of view [60].

However, the Schnitzler syndrome shares many features with genetically determined auto-inflammatory syndromes:

- The recurrent fever of unknown cause;

- The peculiar eruption, characterized pathologically by a neutrophilic infiltrate very similar to the one observed in the auto-inflammatory cryopyrinopathies (CINCA/NOMID syndrome, Muckle-Wells syndrome and familial cold-urticaria), namely a neutrophilc urticarial dermatosis [9];

- A significant increase of neutrophils in blood and tissues, not otherwise explained;

- An increased IL-1beta production by LPS-stimulated peripheral blood monocytes [61,62];

- Elevated free circulating IL-18 levels, a cytokine produced by the inflammasome [63];

- A genetic predisposition involving an activating *NLRP3 *mutation, the gene involved in the cryopyrinopathies, which was demonstrated in one patient [64];

- A spectacular response to the IL-1 inhibitor anakinra [23,65], within hours after the first injection, just as the one observed in the cryopyrinopathies, suggesting a direct pathogenic effect of IL-1.

Thus, the Schnitzler syndrome is probably an acquired auto-inflammatory syndrome. The monoclonal IgM component could then either be the consequence of a specific cytokinic activation pathway or it could be directly involved in the pathogenesis *via *a particular biological activity, as for example an agonist activity on one of the IL-1 receptors or by fixing IL-1 and lowering its clearance without altering its biological activity or by inhibiting the natural occurring IL-1 antagonist. This hypothesis is currently tested in an international clinical study http://clinicaltrials.gov/ct2/show/NCT00933296. If the latter hypothesis is confirmed, the Schnitzler syndrome would be an extrinsic inflammasopathy.

### Treatment

Many treatments used in the Schnitzler syndrome are summarized in Table 2. Among the numerous drugs, including anti-inflammatory and immunosuppressive drugs, which have been tried, none could induce remission for a long time of all symptoms [reviewed in [6]]. Peflacine, a quinolone antibiotic could induce almost complete remission in some patients, though its mechanism of action remains largely unknown [66,67]. Thus, the Schnitzler syndrome was a very difficult-to-treat syndrome and patients had to learn how to deal on a daily basis with symptoms that could not be correctly relieved. This was changed with the use of the IL-1 receptor antagonist anakinra. The first short note about its efficiency was published by Martinez-Taboada et al in 2005 [68] and it was subsequently confirmed by many reports and this experience is shared by many physicians treating patients with a Schnitzler syndrome [23,57,64,69,70]. Anakinra, an IL-1 receptor antagonist relieves all symptoms within hours after the first injection. Patients recover a health-state that they did not know for years. Anakinra has a short half-live of about 6 hours and daily injections are necessary. In this author's experience, if a patient omits an injection, symptoms, most notably fever, pain and the skin rash, usually recur between the 35^th ^and the 45^th ^hour after the last injection. A few patients seem to be relatively well controlled with an injection every other day (J.-P. Fermand, personal communication). Anakinra proved to be the first efficient drug to treat this syndrome. We do not yet have experience with other IL-1-blocking agents as IL-1 trap or canakinumab. The main side-effects and the contra-indications of anakinra are summarized in the Additional file 1: table S1. Injection-site reactions are frequent and sometimes severe, and can be a real concern. The neutrophil count needs to be monitored. As of today, it is the only treatment which showed regular efficiency in this syndrome.

**Table 2:** Treatments of patients with the Schnitzler syndrome before the use of anakinra

Treatment	Comment
Steroids	Suspensive, but usually requiring a high daily dosis (>40 mg equivalent of prednisolone) to achieve improvement

Non steroid anti-inflammatory drugs, most notably ibuprofen	Transient amelioration of fever and pain

Immunosuppressive drugs, including methotrexate, azathioprine, cyclophosphamide	Usually ineffective

Colchicine, dapsone	Transient improvement

Thalidomide	Anecdotic reports of efficiency

TNF-blocking agents	Ineffective

Immunoabsorption	Only a single report

Intravenous immunoglobulins	Ineffective

Rituximab	Ineffective

Anti-histamines	Usually ineffective, even on the skin rash

Phototherapy	Transient amelioration of skin rash

Peflacine	Efficient in some patients; recurrence if treatment is stopped.

Anakinra	Complete and sustained remission of all symptoms; recurrence if treatment is stopped

## Conclusion

This author considers that the Schnitzler syndrome is the paradigm of a late-onset acquired auto-inflammatory syndrome [71]. Though the term "auto-inflammatory disease" is as yet restricted to diseases with mendelian inheritance, the Schnitzler syndrome obviously shares many clinical, biological and therapeutical aspects with this group of diseases. Though there is no definite proof of its precise pathogenesis, it should therefore be considered as an acquired disease involving abnormal stimulation of the innate immune system, which can be completely reversed by the IL-1 receptor antagonist anakinra. It clearly expands our view of this group of rare genetic diseases and makes the concept of auto-inflammation relevant to polygenic acquired diseases.

## Competing interests

The author declares that they have no competing interests.

## Supplementary Material

Additional file 1 **Table S1**. Main side-effects and contra-indications of anakinra. Click here for file
