# Supplementary material for: The Schnitzler syndrome
Source: Orphanet J Rare Dis. 2010 Dec 8;5:38. doi: 10.1186/1750-1172-5-38 (PMC3018454; doi:10.1186/1750-1172-5-38)
Supplement: Additional file 1 — Table S1. Main side-effects and contra-indications of anakinra. [file 1750-1172-5-38-S1.DOC]

**Table S1** Main side-effects and contra-indications of anakinra

| Injection-site reaction +++ (50 to 80% of patients) |
| --- |
| Headache (> 10%), nausea, rhinitis, diarrhea, abdominal pain |
| Rare: neutropenia (2,4%), serious infections (2%, skin, lungs, bones) |
| Dermatological side-effects: urticarial and maculo-papular exanthema, interstitial granulomatous dermatitis, Wells phenomenon, psoriasis |
| Contra-indication: allergy, renal clearance < 30ml/min |
